# Antiviral Type I and Type III Interferon Responses in the Central Nervous System

**DOI:** 10.3390/v5030834

**Published:** 2013-03-15

**Authors:** Frédéric Sorgeloos, Marguerite Kreit, Pascale Hermant, Cécile Lardinois, Thomas Michiels

**Affiliations:** Université catholique de Louvain, de Duve Institute, VIRO B1.74.07, 74 avenue Hippocrate, B-1200, Brussels, Belgium; E-Mails: frederic.sorgeloos@uclouvain.be (F.S.); marguerite.kreit@uclouvain.be (M.K.); pascale.hermant@uclouvain.be (P.H.); cecile.lardinois@uclouvain.be (C.L.)

**Keywords:** interferon alpha/beta, interferon lambda, interferon-stimulated gene (ISG), neuron, astrocyte, neurotropic virus, axonal transport

## Abstract

The central nervous system (CNS) harbors highly differentiated cells, such as neurons that are essential to coordinate the functions of complex organisms. This organ is partly protected by the blood-brain barrier (BBB) from toxic substances and pathogens carried in the bloodstream. Yet, neurotropic viruses can reach the CNS either by crossing the BBB after viremia, or by exploiting motile infected cells as Trojan horses, or by using axonal transport. Type I and type III interferons (IFNs) are cytokines that are critical to control early steps of viral infections. Deficiencies in the IFN pathway have been associated with fatal viral encephalitis both in humans and mice. Therefore, the IFN system provides an essential protection of the CNS against viral infections. Yet, basal activity of the IFN system appears to be low within the CNS, likely owing to the toxicity of IFN to this organ. Moreover, after viral infection, neurons and oligodendrocytes were reported to be relatively poor IFN producers and appear to keep some susceptibility to neurotropic viruses, even in the presence of IFN. This review addresses some trends and recent developments concerning the role of type I and type III IFNs in: i) preventing neuroinvasion and infection of CNS cells; ii) the identity of IFN-producing cells in the CNS; iii) the antiviral activity of ISGs; and iv) the activity of viral proteins of neurotropic viruses that target the IFN pathway.

## 1. Antiviral IFN Responses

Interferons (IFNs) were discovered about 50 years ago, as soluble factors produced by chicken cells of the chorio-allantoic membranes after contact with influenza virus, which interfered with subsequent viral infection. This review will focus on type I and type III IFNs, also known as IFNs-α/β and IFN-λ, respectively and further referred to as IFNs in this review. These IFNs can be produced by many cell types and primarily act as antiviral cytokines, although they also exhibit cytostatic activities and help to activate and shape the adaptive immune response. In contrast, type II IFN (or IFN-γ is produced by cells of the immune system such as macrophages, T cells and natural killer cells. IFN-γ primarily acts as an immunomodulatory cytokine that notably contributes to T cell polarity and activates cellular immunity. It also displays direct antiviral activity and has been shown to be a critical mediator of neuron protection against Sindbis virus [1].

**Figure 1:**
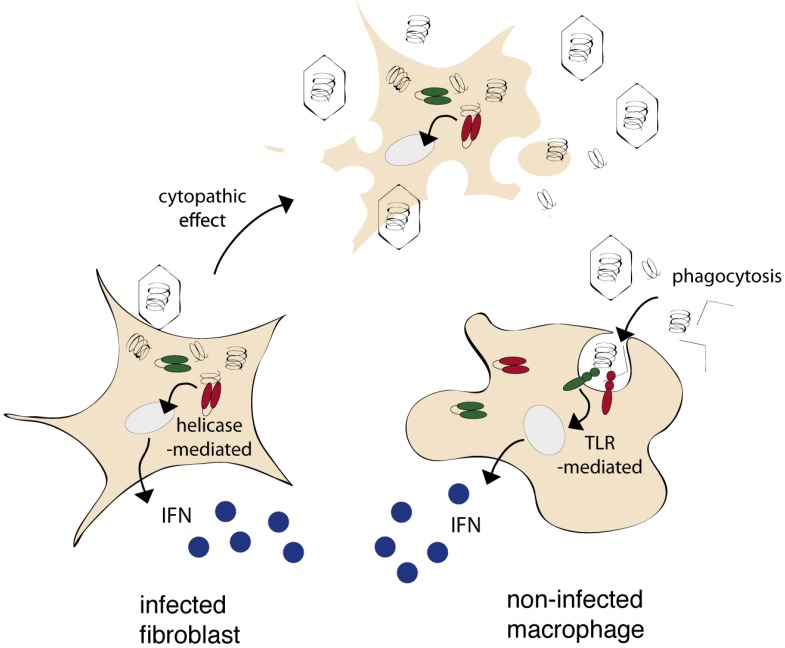
Infected and non-infected cells can produce IFN, using distinct pattern recognition receptors. Most cells express RIG-like helicases (RIG-I or MDA-5) that sense nucleic acids of viral origin in the cytoplasm and thus trigger IFN production by infected cells. Some cells, and particularly phagocytic cells, express TLRs that sense extracellular danger and pathogen-associated molecular patterns from the extracellular milieu. TLRs thus enable non-infected cells to sense viral components released by neighboring cells.

Cells can express two types of sensors, known as “pattern recognition receptors” (PRRs), that act to detect microbial or viral components present either in the cytoplasm or in the extracellular milieu, and activate a signal transduction pathway which culminates in the expression and the secretion of IFNs (Figure 1) (reviewed in [2]). Receptors of the RIG-I helicase family act to detect intracytoplasmic nucleic acids of viral origin and are thus expected to trigger IFN expression by infected cells. Receptors of the Toll-like receptor family (TLRs) are expressed at the cell surface or in the endosomal compartment and thus enable non-infected cells to sense viral components from the extracellular environment (Figure 1). TLRs can be expressed by many cell types but are usually more strongly expressed in antigen presenting cells such as dendritic cells or macrophages (See [3] for TLR expression in the CNS).

After secretion, IFNs bind to their cognate receptor and induce the expression of hundreds of genes referred to as “interferon-stimulated genes” (ISGs). These genes encode proteins such as Mx, PKR, OAS, or IFIT1/2 that enhance the resistance of cells toward a potential viral infection. Importantly, some ISGs encode signaling molecules involved in the IFN production or response pathway. They thus create a positive feedback loop aimed to boost IFN responses as infection develops.

## 2. Critical Importance of the IFN Response Against Neurotropic Virus Infection

The critical importance of IFN to restrict viral infections became obvious after the generation of mice deficient for the IFNAR-I subunit of the type I IFN receptor [4]. These mice turned out to be remarkably susceptible to many viral infections, including viral infections of the CNS (Table 1). A noticeable case is that of Sindbis virus for which LD_50_ values were 10^6^-fold lower in IFNAR-I KO mice than in wild-type mice. This extreme susceptibility of KO mice correlated with increased viral load in the CNS [5]. Although IFN is mostly known to be protective against RNA virus infection, it was also shown to protect the CNS against DNA viruses. For instance, after ocular infection, growth of attenuated Herpes virus mutants in the eye and in trigeminal ganglia was increased by more than 1000-fold in IFNAR-KO mice [6]. More recently, the importance of the interferon response against neurotropic viral infection in humans was evidenced by the discovery that several cases of fatal herpes encephalitis in newborns were associated with genetic deficiencies in genes encoding signal transduction factors of the IFN pathway, such as TANK-binding kinase 1 (TBK-1), Toll-interleukin-1 receptor domain-containing adaptor-inducing beta interferon (TRIF), TLR3, unc93b or tumor necrosis factor receptor-associated factor 3 (TRAF3) [7,8,9,10,11] (reviewed in [12]).

**Table 1:** Infection of type I IFN receptor (IFNAR-I)-deficient mice.

Virus	Family	Observation	Ref
Lassa fever virus	Arenaviridae	Increased viral load and morbidity, modified tropism	[13]
Borna disease virus	Bornaviridae	Switch from transcription to replication	[14]
Hantaan virus	Bunyaviridae	Increased neurovirulence	[15]
Dugbe virus	Bunyaviridae	Increased neurovirulence	[16]
Crimean–Congo hemorrhagic fever virus	Bunyaviridae	Increased viral load and neurovirulence, modified tropism	[17]
La Crosse virus	Bunyaviridae	Increased neurovirulence	[18]
Schmallenberg virus	Bunyaviridae	Increased viral load and morbidity, modified tropism	[19]
Mouse Hepatitis virus	Coronaviridae	Increased viral load and neurovirulence, modified tropism	[20]
West Nile virus	Flaviviridae	Increased viral load and neurovirulence, modified tropism	[21]
Murray Valley encephalitis virus	Flaviviridae	Increased viral load and neurovirulence	[22]
Dengue virus	Flaviviridae	No clear effect of type I IFN	[23]
Herpes simplex virus 1	Herpesviridae	Increased viral load	[6]
Influenza A virus	Orthomyxoviridae	Increased viral load in CNS	[24]
Thogoto virus	Orthomyxoviridae	Increased viral load in CNS, modified tropism	[25]
Measles virus	Paramyxoviridae	Increased neurovirulence	[26]
Hendra virus	Paramyxoviridae	Increased viral load and neurovirulence, modified tropism	[27]
Nipah virus	Paramyxoviridae	Increased viral load and neurovirulence, modified tropism	[27]
Poliomyelitis virus	Picornaviridae	Increased neurovirulence, modified tropism	[28]
Theiler’s virus	Picornaviridae	Increased viral load and neurovirulence	[29,30]
Reovirus	Reoviridae	Increased viral load and neurovirulence, modified tropism	[31]
Vesicular stomatitis virus	Rhabdoviridae	Increased viral load and neurovirulence	[4,32]
Rabies virus	Rhabdoviridae	Increased neurovirulence	[33]
Sindbis virus	Togaviridae	Increased viral load and neurovirulence, modified tropism	[5]
Venezuelan equine encephalitis virus	Togaviridae	Increased neurovirulence	[34]
Chikungunya virus	Togaviridae	Increased viral load and neurovirulence, modified tropism	[35]
Eastern equine encephalitis virus	Togaviridae	Increased neurovirulence	[36]
Semliki Forest virus	Togaviridae	Increased viral load and neurovirulence, modified tropism	[37,38]

## 3. Low Endogenous IFN Response in the CNS and IFN Neurotoxicity

Early work showed that IFNs are constitutively expressed at low levels in mice and humans and may therefore exert homeostatic functions [39,40]. It was suggested that such constitutive IFNs maintain cells ready to switch on rapid and efficient IFN responses [41]. However, basal ISG mRNA levels detected in the CNS appear to be lower than those detected in peripheral tissues [28]. The low activation of ISGs in the CNS likely stems from the inability of IFNs produced in the periphery, notably in lymphoid or mucosal tissues, to cross the blood-brain barrier. The low basal IFN activity in the CNS has likely been evolutionary favored given the reported neurotoxicity of IFN. Indeed, neurological and neuropsychiatric adverse effects like depression, cognitive dysfunction and disorientation have been observed after high-dose IFN-α treatment (reviewed in [42]). The particular sensitivity of the CNS to high IFN doses is particularly exemplified in the case of the Aicardi-Goutières syndrome, a progressive encephalopathy which develops in patients that overexpress endogenous IFN genes [43]. Mutations responsible for this disease have been found in genes coding for various enzymes such as exo- and endonucleases that are believed to control the intracellular pool of aberrant nucleic acid species (single-stranded DNA, dsRNA, triphosphorylated RNA...) known to activate RIG-like helicases and/or TLRs [44,45,46]. High levels of IFN-α can be measured in both the serum and the cerebro-spinal fluid of these patients, but the most dramatic manifestations of the disease appear in the CNS, underlining the particular sensitivity of this organ to IFN.

## 4. Antiviral Activity of IFN-λ in the CNS

Type III IFNs were discovered about 10 years ago by two independent groups [47,48]. The type III IFN family comprises three subtypes, IFN-λ1, IFN-λ2 and IFN-λ3, also named IL29, IL28A and IL28B respectively. In the mouse, IFN-λ1 is a pseudogene, whereas all 3 genes are expressed in humans [47,48,49]. Type III IFNs signal through a receptor distinct from that of type I IFNs [47,48,50] but trigger the same signal transduction pathway downstream of the receptor and upregulate the same group of ISGs [49,51,52,53]. Nevertheless, the range of cells that respond to type I and type III IFNs differs. While the type I IFN receptor can be expressed by most cell types, the type III IFN receptor appears to be preferentially expressed by epithelial cells [54].

In the CNS, type III IFNs seem to be less expressed than type I IFNs in response to viral infection. Upon infection with Mouse hepatitis virus (MHV) or Lactate dehydrogenase-elevating virus (LDV), both IFN-α and IFN-β mRNA were easily detected in the brain and liver of infected mice. In contrast, low levels of IFN-λ mRNA were detected in the brain of these mice, while expression of this IFN was readily detected in the liver [54]. Some IFN-λ expression was detected in primary neurons and in primary astrocytes after poly I:C stimulation [55]. These data suggest that neurons and astrocytes might express some levels of IFN-λ. However, more studies are required to confirm the relevance of this observation *in vivo*.

Various cell types of the CNS, including oligodendrocytes, astrocytes and neurons, were reported to respond to IFN produced upon viral infection. However, very little is known about the specific responsiveness of CNS cells to IFN-λ. Quantitative RT-PCR data show an overall weak expression of the IL28R-α subunit of the IFN-λ receptor in the CNS as compared to other tissues [54]. Sommereyns *et al*. used *in vivo* expression of IFN-λ3 to identify the cells that can respond to circulating IFN produced by muscle cells in the periphery. In this experiment, the Mx1 protein, used as a marker of the IFN response, was detected only in the epithelial cells of choroid plexus and in few meningeal cells. These data are consistent with the epithelial specificity of the IFN-λ response. It is noteworthy that in this experiment, the access of IFN to the brain parenchyma was restricted by the BBB and only endothelial and choroid plexus cells were expected to be reached by circulating IFN.

It was recently observed that IFN-λ can inhibit HSV-1 infection in primary human astrocytes [53]. Further experiments are thus required to address the identity of cells that respond to IFN-λ *in vivo*, after CNS infection and to get more insights about the relative contributions of IFN-λ and IFN-α/β in the resistance against neurotropic viruses. Mx1-positive congenic mice that lack either the type I or the type III IFN receptor might provide adequate tools to tackle these questions [56]. However, given the high susceptibility of type I IFN receptor KO mice (which still have a type III IFN response) toward many neurotropic viruses (see Table 1) and the relatively low expression of the IFN-λ receptor in the CNS, we anticipate that the contribution of IFN-λ in the protection of the CNS against viral infection will be modest.

## 5. IFN Producing Cells in the CNS

In peripheral tissues, plasmacytoid dendritic cells (pDCs) are recognized as major IFN-producing cells in the context of a viral infection [57]. For instance, pDC-produced IFN was found to be instrumental in resistance against coronavirus infection [58]. However, other immune cells as well as resident cells may substantially contribute to IFN production as well. Under physiological conditions, the CNS fails to contain pDCs but microglial cells and perivascular dendritic cells are expected to serve as phagocytic cells that initiate immune responses. *In vitro*, the various CNS cell types can produce IFN, including neurons. The latter cells were reported to produce IFN in a TLR-3-dependent manner, after rabies or West-Nile virus infection [59,60].

Delhaye *et al*. used *in situ* hybridization and immunohistochemistry to characterize *in vivo* IFN-producing cells, after infection with two neurotropic viruses that infect mostly neurons: La Crosse virus *(bunyaviridae)* and the GDVII neurovirulent strain of Theiler's virus *(picornaviridae)* [61]. These authors showed that: i) resident CNS cells rather than infiltrating inflammatory cells were mostly responsible for IFN production; ii) about 16% of IFN-producing cells corresponded to neurons. However, only 3% of infected neurons appeared to produce IFN which suggests that neurons produce IFN in a highly controlled fashion.

**Figure 2:**
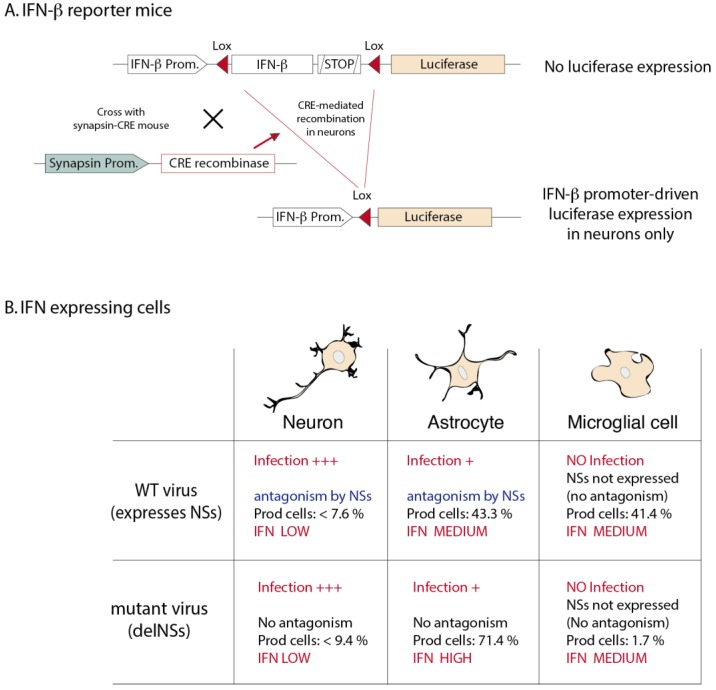
IFN-β reporter mice and IFN-β producing cells after LaCrosse virus infection. (**A**) Knock-in reporter mice. The IFN-β ORF, followed by a polyadenylation signal is floxed (Lox sites are represented by red arrowheads). A firefly luciferase ORF present downstream of the floxed region can be transcribed by the IFN-β promoter after CRE-mediated recombination. When these mice are crossed with mice that express CRE in a cell-specific fashion, luciferase expression, driven by the IFN-β promoter, will be restricted to that specific cell type. The example of the neuron-specific synapsin promoter is shown (adapted from Lienenklaus *et al*., [63]). (**B**) IFN-β expressing cells in La Crosse virus infected brains (adapted from Kallfass *et al*., [62]). A wild-type (WT) strain of La Crosse virus was used as well as the delNSs mutant, lacking the IFN antagonist non-structural protein NSs. Although neurons were heavily infected, very few produced IFN-β (luciferase), suggesting that IFN production by neurons is strictly regulated. In astrocytes, NSs expression appears to block IFN expression efficiently. Microglial cells are not infected but produce IFN, likely in a TLR-dependent way.

A recent study by Kallfass *et al*. elegantly readdressed the question using reporter mice that allow both immunostaining and quantitative luciferase assays [62]. In these reporter mice, the luciferase ORF was substituted for the IFN-β ORF and is thus transcriptionally dependent on the genuine IFN-β promoter. Interestingly, a floxed "stop cassette" is inserted between the IFN-β promoter and the luciferase gene so that IFN-β-dependent luciferase expression only occurs in specific cell types after crossing the reporter mice with mice expressing the CRE recombinase in the cells of interest (Figure 2) [63]. Using these mice and La Crosse virus infection, Kallfass *et al*. showed that astrocytes and microglial cells/macrophages accounted for 43% and 41% of luciferase (IFN-β) expression respectively, although viral antigen-positive cells were mostly neurons. Viral antigen was present in few astrocytes but not in microglial cells. Interestingly, in mice infected with a mutant virus which does not express the IFN-antagonist NSs protein [18], astrocytes accounted for more than 70% of luciferase activity and the contribution of macrophages became marginal (1.7%) [62]. Taken together, these experiments suggest that (Figure 2): i) resident cells and not specialized immune cells are indeed the main IFN producers in the CNS; ii) only few infected neurons do produce IFN; iii) infected astrocytes produce IFN but this IFN production can be antagonized by the NSs protein; iv) non-infected microglial cells produce IFN, likely by a TLR-dependent pathway. The contribution of microglial cells becomes much more important when IFN production is inhibited in infected cells by the non-structural protein NSs.

Interferon-producing cells were also studied after infection of the CNS by the neurotropic Mouse hepatitis virus (MHV). IFN was mostly produced by macrophages and/or microglial cells. In this case however, IFN production was dependent on the cytoplasmic helicase MDA-5, suggesting that IFN was produced by infected cells [64].

As is the case in neurons, IFN production may be restricted in oligodendrocytes. A recent study showed that microglial but not oligodendroglial cells isolated from mice infected with MHV expressed detectable IFN-β levels although both cell types were infected by the virus. Low basal expression of sensors and of signaling molecules in oligodendrocytes was proposed to limit the rapid responsiveness of these cells [65].

## 6. Control of Neuroinvasion by IFN

Some neurotropic viruses access the central nervous system (CNS) via the olfactory pathway. They infect the olfactory sensory neurons present in the nasal mucosa and then reach the olfactory bulb. Using conditional knock-out mice deficient for IFNAR-I expression in neural tissues, Detje *et al*. showed that vesicular stomatitis virus (VSV) spread from the olfactory bulb to the entire CNS was efficiently controlled by a local IFN response occurring at the level of the glomerular layer of the olfactory bulb [32,66]. 

**Figure 3:**
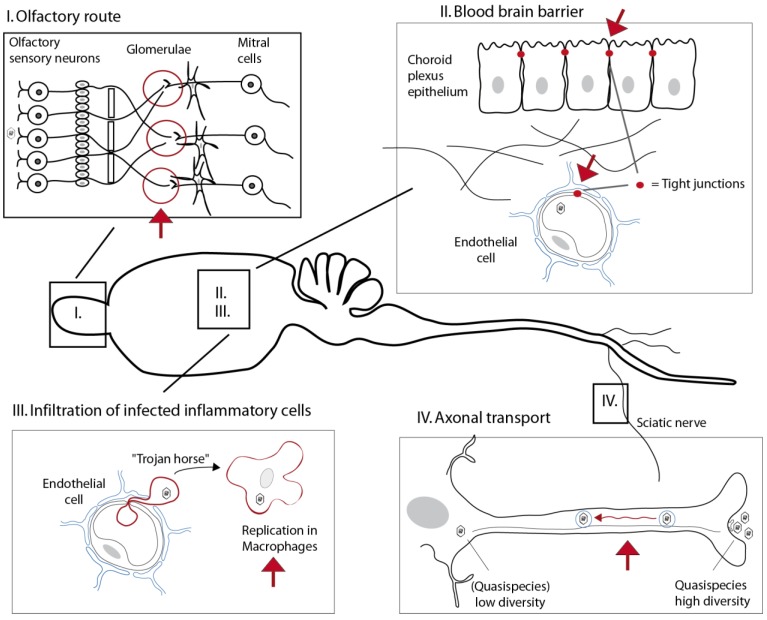
**Expected effects of IFN on neuroinvasion pathways.** Viruses can reach the CNS by the olfactory route (**I**), via the blood-brain barrier (**II**), by infecting infiltrating cells (Trojan horse strategy) (**III**), or by using axonal transport (**IV**). (**I**) In the olfactory pathway, IFN was found to limit viral spread of VSV from the glomerulae that connect olfactory neurons, mitral cells and some periglomerular cells [66]. (**II**) The blood-brain barrier is tightened by tight junctions formed by capillary endothelial cells and between adjacent epithelial cells of the choroid plexus. Epithelial cells of the choroid plexus strongly respond to circulating IFN-λ and endothelial cells respond to circulating IFN-α/β. Type I and type III IFNs are thus believed to concur to protect BBB-forming cells [54]. (**III**) Type I IFN produced in the periphery is expected to limit neuroinvasion via Trojan horses by controlling viral replication in the cells that might infiltrate the CNS. (**IV**) It is still unclear to what extent IFN can control axonal transport. It was reported that IFN acts to restrict the diversity of quasispecies during progression in the sciatic nerve [70].

Most neurotropic viruses, however, infect a peripheral site before they access the central nervous system. To cross the blood-brain barrier, viruses might either take advantage of local damages in this barrier or infect cells that form the barrier, i.e. endothelial cells or epithelial cells of the choroid plexus. Alternative options are to infect immune cells that infiltrate the CNS (“Trojan horse” strategy) or to circumvent the BBB by using axonal transport (Figure 3).

Peripheral infection is expected to induce the secretion of IFN that may act in a systemic fashion to limit neuroinvasion. For example, poliovirus first infects the digestive tract before accessing the CNS. It has been shown that transgenic mice expressing the human poliovirus receptor but lacking the type I IFN receptor are much more susceptible to fatal CNS infection with poliovirus than IFN-competent mice [67]. Although poliovirus can use axonal transport, it is not known in this model, which pathway was followed by poliovirus to infect the CNS in the IFN receptor-deficient mice. IFN produced after intramuscular inoculation of mice with rabies virus was also shown to slow down CNS invasion and to delay mortality, even in conditional KO mice that were deficient for IFNAR-I only in cells of neuroepithelial origin [68].

IFN produced in the periphery does not cross the BBB efficiently [32]. However, cells that form the BBB do respond to circulating IFN: endothelial cells readily respond to circulating IFN-α/β and epithelial cells of the choroid plexus respond more strongly to IFN-λ [54] (and unpublished observations). This suggests that type I and type III IFNs concur to limit neuroinvasion via infection of cells that form the BBB. However, data are still lacking to circumstantiate this view.

IFN expressed in the periphery might also affect axonal transport. It was found that the neuroinvasion step represents a major bottleneck in the spread of the viral quasispecies formed by poliovirus. Vignuzzi *et al*. showed that heterogeneity of the viral population in the periphery was a prerequisite for neuroinvasion by poliovirus unless type I IFN response was compromised [69]. Lancaster *et al*. showed that more viral pools progressed from the lower to the upper segment of the sciatic nerve of poliovirus when the type I IFN receptor was lacking, suggesting that the interferon response could modulate the efficiency of the axonal transport of viruses [70]. How IFN limits transport and/or quasispecies diversity is another open question that warrants future work.

## 7. IFN Responding Cells

*In vivo*, the various CNS cell types were reported to have the capacity to respond to IFN-I and therefore to express ISGs [61,71,72]. Neurons are again a particular case. For instance, their capacity to express MHC class-I molecules (inducible by both type I and type II IFNs) has been debated. It has been shown that class-I MHC molecules were expressed on neurons in a type I IFN-dependent fashion, after TMEV infection [71]. Moreover, neurons infected with borna disease virus were shown to be targeted efficiently by cytolytic T cells, suggesting that MHC class-I molecules can be functional on neurons [73]. However, Neumann *et al*. previously suggested that MHC class-I molecules expression of neurons, allowing killing of the cells, was restricted and only occurred after irreversible damage of the neurons [74], which fits with the view that non-cytolytic responses may be favored in neurons [75].

A possible explanation for the conflicting results obtained with neurons is that different neuronal populations might strongly differ in their responsiveness to IFN. Such a striking difference in responsiveness was observed in transgenic mice constitutively expressing IFN-α in the CNS. Interestingly, these mice exhibited a strong Mx expression in CA1 and CA2, but not in CA3 neurons of the hippocampus [14]. Recently, it was also observed that dorsal root ganglionic neurons (although not from the CNS) poorly responded to type I IFN treatment and favored autophagy as a mechanism for clearance of herpes virus infection [76].

It can also be speculated that, in view of the low basal expression of IFN and ISGs in the CNS, some neuron populations express too low STAT-1 levels to mount an efficient antiviral response.

As neurons, oligodendroglial cells were recently reported to be poorly reactive to interferon as compared to microglial cells. Oligodendrocytes isolated from mice were more susceptible than microglial cells and showed delayed ISG expression. Again, it is anticipated that the low levels of signal transduction molecules present in these cells in non-inflammatory conditions might hamper a prompt IFN response in these cells [65].

## 8. Interferon-Stimulated Genes

**Figure 4:**
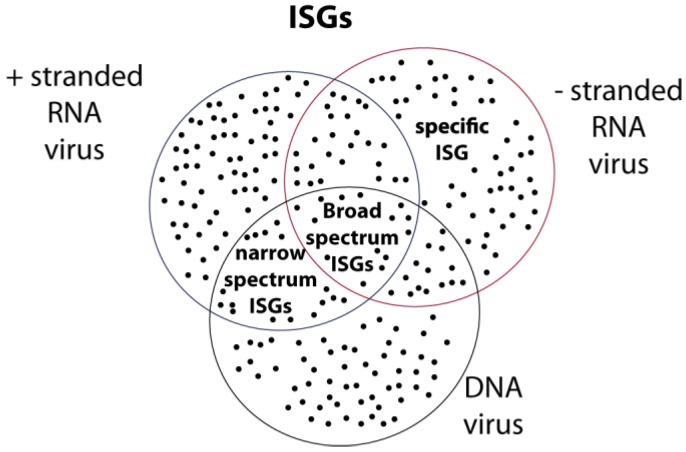
**ISGs act in combination.** i) The antiviral activity of ISGs appears to be the combination of many individual contributions. ii) As many ISGs display some specificity in their antiviral action, each virus species is likely controlled by a unique combination of many ISGs.

Hundreds of ISGs have been identified in cells treated with type I or type III IFN or after viral infection of the CNS [77,78,79,80]. Until recently, the mode of action of a relatively limited set of ISGs displaying antiviral activity has been characterized (for review see [81,82]). Some of these ISGs exhibit specificity for their target virus. An example of such ISGs is the Mx family of proteins that mostly target RNA viruses and were named after they were discovered to confer resistance to myxoviruses “Mx” [83]. Other examples of ISGs that exhibit specificity for their target viruses include the APOBEC3G editing enzyme that was discovered as a restriction factor of human immunodeficiency virus [84] or the promyelocytic leukemia proteins (PML) which can target DNA and RNA viruses but display specificity according to the isoform that is expressed [85]. Other ISGs act against a broader range of viruses. These includes ISGs coding for RIG-like helicases which are involved in a positive feedback loop of IFN production and therefore enhance the expression of many other ISGs. PKR also acts on a broad range of viruses as it is both an inducer and an effector of the IFN response [81]. Interestingly, two recent broad screens allowed the identification of a series of additional ISGs that interfere with viral replication when expressed ectopically [86,87]. Together, these studies have underscored the specificity and the cumulative activity of ISGs. On one hand, the expression of a single ISG can impact the replication of a given range of viruses. On the other hand, multiple ISGs can impact the replication of a single virus. Therefore, it is expected that distinct ISG combinations are instrumental in the control of distinct viruses (Figure 4).

Until now, little has been described about the potential histospecific activity of some ISGs. In that respect, Fensterl *et al*. recently demonstrated the importance of ifit2, but not of the related ifit1, against VSV infection of the CNS. Ifit-2 KO mice showed a dramatically increase in the viral load in the brain but not in other organs [88], suggesting that this ISG might specifically act in the context of the CNS.

Additional CNS-specific ISGs might be discovered in the future, as targets of antagonist proteins produced by highly neurotropic viruses.

## 9. Antagonism of the IFN Response by Neurotropic Viruses

Most neurotropic viruses encode one or more proteins aimed at interfering with the IFN pathway. These proteins are often multifunctional and sometimes interfere with different targets of the same pathway. 

As an example, rabies virus is a highly neurotropic virus responsible for a fatal disease in a wide range of animals and in humans. The P phosphoprotein is one of the five proteins encoded by the virus. Besides its involvement in viral RNA synthesis as a cofactor of the polymerase, the P protein of rabies virus is a paradigm of non-structural protein interfering with IFN induction, IFN signaling as well as IFN-induced antiviral effectors.

The P phosphoprotein was shown to prevent the phosphorylation of IRF3 by TBK1 so that IRF3 dimerization and transcriptional activation of IFN genes is inhibited in infected cells [89]. P was also shown to interfere with IFN signaling. It specifically interacts with tyrosine-phosphorylated STAT1 and STAT2 and sequesters these proteins in the cytoplasm, thus preventing JAK-STAT signaling and transcription of ISGs. Moreover, interaction of P with STATs also blocked STAT1 and ISGF3 binding to the promoter of IFN responsive genes [90,91,92]. Interestingly, mutant P proteins that lost either activity (IRF-3 activation or STAT1 inhibition) impaired rabies virus neurovirulence, suggesting additive activities of the various functions [93,94]. Finally, P protein physically interacts with promyelocytic leukemia proteins (PML) to counteract the activity of this family of IFN-inducible proteins [95,96,97] (Table 2). 

The phosphoprotein of rabies virus thus offers a typical example of multifunctional protein that evolved to interfere with the various steps of the IFN pathway: IFN production, IFN response, and effectors activity. It is noteworthy that the phosphoproteins of the distantly related neurotropic Nipah and Hendravirus (or the V and W proteins derived from the same gene by editing) also interfere with various steps of the IFN pathway, yet using additional mechanisms (Table 2).

**Table 2:** Inhibition of the IFN pathway by rabies, Hendra and Nipah virus phosphoproteins products.

Virus	Family	Protein	Mechanism	References
Rabies virus	Rhabdoviridae	P	Inhibition of IRF3 phosphorylation	[89]
			by TBK1	
			Sequestration of STAT1/2 in the cytoplasm	[90,91]
			Inhibition of ISGF3 binding to promoter	[92]
			Interaction with PML	[95,96]
Hendra and Nipah viruses	Paramyxoviridae	V	Inhibition of MDA-5	[98]
				[99]
		V	Lgp2 + RIG-I	[100]
		W	Inhibition of TLR3 signaling via TRIF	[101]
		P, V, W	Inhibition of STAT-1 phosphorylation	[102,103,104,105]

Another striking example of multifunctional proteins that evolved to inhibit the IFN pathway is given by positive-stranded RNA virus- (and retrovirus-) encoded proteases. These enzymes primarily act to process polyproteins encoded by the virus. Yet, they evolved to cleave several host proteins involved in cell defences. For example, 2A and 3C proteases of poliovirus not only induce a shut-off of host protein synthesis by cleaving the eIF4G eukaryotic translation initiation factor but also cleave TRIF, RIG-I, and the p65 subunit of NF-κB which participates to activate IFN gene transcription (Table 3). In evolutionary terms, it is interesting to note that other neurotropic picornaviruses like Theiler's murine encephalomyelitis virus (TMEV) or encephalomyocarditis virus (EMCV) evolved to encode a non-structural protein, L, that lacks protease activity but targets very similar functions as those targeted by polio or rhinovirus proteases [30,106,107,108].

In conclusion, neurotropic viruses acquired multiple mechanisms devoted to evade the IFN pathway, which confirms the critical importance of this pathway in the infection of the CNS. On the one hand, many viral proteins display multifunctionality and target several host defence pathways. On the other hand, viruses often develop more than one antagonist to target a single pathway. This strategy likely limits the possibility of the host cell to control viral infection by developing new weapons in a war escalation attempt. Yet, viruses seldom provoke complete inhibition of the IFN pathway *in vivo*. By doing so, they limit the risk of becoming too virulent and thus to prevent virus transmission due to premature death of the host.

It is worth noting that most activities that were uncovered in the case of neurotropic viruses do not point to pathways that would be specific for the CNS environment. It is unclear whether IFN antagonist proteins produced by neurotropic viruses are more important during the initial phase of the infection which often happens in the periphery or during the neuroinvasion phase.

**Table 3:** Examples of proteases from neurotropic positive-stranded RNA viruses and from retroviruses that interfere with the IFN pathway.

Virus	Family	Protease	Mechanism	References
Encephalo-myocarditis virus	Picornaviridae	3C	Cleavage of RIG-I	[109]
Coxsackievirus	Picornaviridae	3C	Cleavage of MAVS and TRIF	[110]
Poliovirus	Picornaviridae	2A	Cleavage of ISGs	[111]
Poliovirus	Picornaviridae	2A	Cleavage of eIF4G	[112]
Poliovirus	Picornaviridae	3C	Cleavage of RIG-I	[113]
Poliovirus	Picornaviridae	3C	Cleavage of eIF5B	[114]
Poliovirus	Picornaviridae	3C	Cleavage of p65-RelA subunit of *NF-kB*	[115]
Enterovirus 71	Picornaviridae	2A	Cleavage of IFNAR1	[116]
Enterovirus 71	Picornaviridae	3C	Sequestration of RIG-I	[117]
Enterovirus 71	Picornaviridae	3C	Cleavage of TRIF	[118]
Dengue virus	Flavivirus	NS2B3	Cleavage of STING	[119,120]
HIV	Retroviridae	Pro	Cleavage of eIF4G	[121]
HIV	Retroviridae	Pro	Sequestration of RIG-I	[122]
Mouse hepatitis virus	Coronaviridae	nsp3	Deubiquitination of TBK1	[123]
Human coronavirus (HCoV)	Coronaviridae	papain-like protease (PLP)	Non-proteolytic disruption of STING-MAVS-TBK1/IKKε complexes	[124]

## 10. Concluding Remarks

There can be no doubt that IFN plays a critical role in the control of viral infections of the CNS, both in mice and humans. To this end, IFN can act at three levels: i) it can limit viral replication in the periphery, before neuroinvasion; ii) it can act at the neuroinvasion step, by protecting the blood-brain barrier or by delaying axonal transport of the virus; and iii) it can act to limit viral spread within the CNS. The involvement of IFN in the periphery has been well documented. In contrast, the role of IFN in axonal transport or in the protection of the BBB requires further studies and it is expected that tools like conditional KO mice lacking an IFN receptor chain in specific cells would be instrumental in such studies. The last step, viral spread within the CNS, has been the focus of some recent studies. The picture that emerges suggests that specific cells of the CNS, namely neurons and oligodendrocytes, have a restricted capacity to produce IFN and to respond to IFN [62,65]. An explanation could be a low basal expression of ISGs in these cells, likely owing to the reported neurotoxicity of IFN. Some ISGs, like RIG-like helicases or STAT-1, participate in a positive feedback loop linking IFN response and IFN production. Cells with low endogenous ISG levels, such as neurons or oligodendrocytes, would thus require a longer exposure to IFN to become IFN producers or to mount an efficient antiviral response. 

IFN-λ has been another focus of many recent studies. However, until now, available data do not suggest a major influence of this IFN type against viral spread within the CNS. IFN-λ was shown to trigger a strong response in epithelial cells of the choroid plexus and might therefore participate in the protection against viruses that would cross the BBB by infecting these cells [54].

Clearly, more studies are needed to answer the many questions that remain concerning the specificity of the IFN response in the CNS. Such studies are however hampered by the need for *in vivo* experiments since the complex relationship between the immune system and the nervous system cannot be assessed with available tools *in vitro*.

Another topic that has much progressed recently is that of ISGs. Large-scale studies have identified a number of ISGs that contribute to the resistance against viruses [86,87]. It is becoming clear that resistance to a specific virus is provided by the combined action of many ISGs that each act on a given virus range. A challenge for the future will be to unravel the mode of action of those ISGs and to understand the basis of their specificity. An open question remains as to whether some ISGs specifically act in CNS cells.

Finally, a major recent progress has been the observation that various factors of the IFN pathway are critically important in humans, against herpes virus encephalitis. The rapid progress of human genetics is expected to fill the gap between the understanding of the IFN response in animal models and in humans.
